# The Expression of Genes Related to Reverse Cholesterol Transport and Leptin Receptor Pathways in Peripheral Blood Mononuclear Cells Are Decreased in Morbid Obesity and Related to Liver Function

**DOI:** 10.3390/ijms25147549

**Published:** 2024-07-09

**Authors:** Carlos Jiménez-Cortegana, Soledad López-Enríquez, Gonzalo Alba, Consuelo Santa-María, Gracia M. Martín-Núñez, Francisco J. Moreno-Ruiz, Sergio Valdés, Sara García-Serrano, Cristina Rodríguez-Díaz, Ailec Ho-Plágaro, María I. Fontalba-Romero, Eduardo García-Fuentes, Lourdes Garrido-Sánchez, Víctor Sánchez-Margalet

**Affiliations:** 1 Department of Medical Biochemistry, Molecular Biology and Immunology, University of Seville Medical School, 41009 Seville, Spain; cjcortegana@gmail.com (C.J.-C.); slopez9@us.es (S.L.-E.); galbaj@us.es (G.A.); margalet@us.es (V.S.-M.); 2 Department of Biochemistry and Molecular Biology, University of Seville Pharmacy School, 41012 Seville, Spain; csm@us.es; 3 Unidad de Gestión Clínica de Endocrinología y Nutrición, Hospital Universitario Virgen de la Victoria, Instituto de Investigación Biomédica de Málaga y Plataforma en Nanomedicina-IBIMA Plataforma BIONAND, 29010 Málaga, Spain; graciamaria_mn@hotmail.com (G.M.M.-N.); lourgarrido@gmail.com (L.G.-S.); 4 Unidad de Gestión Clínica de Cirugía General, Digestiva y Trasplantes, Hospital Regional Universitario de Málaga, Instituto de Investigación Biomédica de Málaga y Plataforma en Nanomedicina-IBIMA Plataforma BIONAND, 29010 Málaga, Spain; javier.morenoruiz@gmail.com; 5 Unidad de Gestión Clínica de Endocrinología y Nutrición, Hospital Regional Universitario de Málaga, Instituto de Investigación Biomédica de Málaga y Plataforma en Nanomedicina-IBIMA Plataforma BIONAND, 29010 Málaga, Spain; sergio.valdes@hotmail.es (S.V.); garciasara79@hotmail.com (S.G.-S.); mariafontalba82@gmail.com (M.I.F.-R.); 6 CIBER de Diabetes y Enfermedades Metabólicas Asociadas (CIBERDEM), Instituto de Salud Carlos III, 29010 Málaga, Spain; 7 Unidad de Gestión Clínica de Aparato Digestivo, Hospital Universitario Virgen de la Victoria, Instituto de Investigación Biomédica de Málaga y Plataforma en Nanomedicina-IBIMA Plataforma BIONAND, 29010 Málaga, Spain; cris.rdrz@gmail.com (C.R.-D.); ailec_hp@hotmail.com (A.H.-P.); 8 CIBER Enfermedades Hepáticas y Digestivas (CIBEREHD), Instituto de Salud Carlos III, 29010 Málaga, Spain; 9 Departamento de Farmacología, Facultad de Medicina, Universidad de Málaga, 29010 Málaga, Spain; 10 CIBER Fisiopatología de la Obesidad y Nutrición (CIBEROBN), Instituto de Salud Carlos III, 29010 Málaga, Spain; 11 Institute of Biomedicine of Seville (IBiS), Hospital Universitario Virgen del Rocío/Virgen Macarena, CSIC, Universidad de Sevilla, 41013 Seville, Spain

**Keywords:** obesity, bariatric surgery, Roux-en-Y gastric bypass, Ob-R, LXRα, ABCA1, ABCG1

## Abstract

Obesity is frequently accompanied by non-alcoholic fatty liver disease (NAFLD). These two diseases are associated with altered lipid metabolism, in which reverse cholesterol transport (LXRα/ABCA1/ABCG1) and leptin response (leptin receptor (Ob-Rb)/Sam68) are involved. The two pathways were evaluated in peripheral blood mononuclear cells (PBMCs) from 86 patients with morbid obesity (MO) before and six months after Roux-en-Y gastric bypass (RYGB) and 38 non-obese subjects. In the LXRα pathway, LXRα, ABCA1, and ABCG1 mRNA expressions were decreased in MO compared to non-obese subjects (*p* < 0.001, respectively). Ob-Rb was decreased (*p* < 0.001), whereas Sam68 was increased (*p* < 0.001) in MO. RYGB did not change mRNA gene expressions. In the MO group, the LXRα pathway (LXRα/ABCA1/ABCG1) negatively correlated with obesity-related variables (weight, body mass index, and hip), inflammation (C-reactive protein), and liver function (alanine-aminotransferase, alkaline phosphatase, and fatty liver index), and positively with serum albumin. In the Ob-R pathway, Ob-Rb and Sam68 negatively correlated with alanine-aminotransferase and positively with albumin. The alteration of LXRα and Ob-R pathways may play an important role in NAFLD development in MO. It is possible that MO patients may require more than 6 months following RYBGB to normalize gene expression related to reverse cholesterol transport or leptin responsiveness.

## 1. Introduction

Obesity represents a significant public health problem in developed countries, with a multitude of obesity-related comorbidities. These include a chronic low-grade inflammatory state, insulin resistance, type 2 diabetes mellitus (T2DM), hypertension, hepatic steatosis, and abnormal lipid metabolism, including decreased high-density lipoprotein (HDL) levels. Bariatric surgical procedures, such as laparoscopic Roux-en-Y gastric bypass (RYGB), are recommended as the most effective treatment for morbid obesity (MO). Furthermore, obesity is also associated with high levels of leptin [1,2]. The action of leptin is mediated by the leptin receptor (Ob-R), a member of the class I type cytokine receptor family [3]. Ob-Rb is the long isoform of the leptin receptor, which allows for complete intracellular signaling. In the pathway of Ob-R signaling, the Src-associated mitosis 68 kDa protein (mostly known as Sam68), a member of the signal transduction and activation of RNA metabolism (STAR) family of RNA-binding proteins, has been previously implicated [4]. Sam68 mediates leptin effects in different diseases [4,5] and is involved in the regulation of hepatic gluconeogenesis [6], which is also associated with leptin effects [7].

Liver X receptor α (LXRα) is an oxysterol-activated nuclear receptor present in different human cells that regulates the expression of genes linked to cholesterol metabolism [8]. Previously, we demonstrated a downregulation of LXRα in visceral adipose tissue from patients with MO [9]. In peripheral blood cells, such as macrophages, this nuclear receptor modulates gene transcription involved in homeostasis cholesterol, such as the ATP-binding cassette (ABC) transporters (ABCA1 and ABCG1), lipogenesis, and the anti-inflammatory response [10]. In previous studies [11,12,13], we have demonstrated their presence in peripheral blood mononuclear cells (PBMCs) and the relevance of LXRα and ABCA1 and ABCG1 transporters in regulating cholesterol homeostasis in monocytes/macrophages and neutrophils. In addition to its pivotal role in cholesterol homeostasis, the nuclear receptor LXRα plays a fundamental role in regulating inflammation in these PBMCs.

Obesity is frequently accompanied by metabolic dysfunction-associated steatotic liver disease (MASLD) and sarcopenia, which are closely intertwined [14]. MASLD, formerly known as non-alcoholic fatty liver disease (NAFLD), is defined as the presence of hepatic steatosis, which is defined as the accumulation of neutral lipids such as triacylglycerol and cholesteryl esters within the liver. In MASLD, the role of metabolic syndrome, obesity, and T2DM is essential. Sarcopenia is defined as the loss of skeletal muscle mass and muscle function. These diseases, obesity, sarcopenia, and MASLD, are associated with an increase in insulin resistance and chronic low-grade inflammation. However, the current therapeutic approach for MO patients with MASLD is to ensure weight loss through bariatric surgery.

It is noteworthy that the Ob-R and LXRα pathways are also associated with MASLD. Leptin plays a critical role in the development of liver fibrosis [15]. Additionally, leptin reduced LXRα protein level and activity in hepatic stellate cells [16]. However, the role of LXRα in the liver is debated, with apparently contradictory results. LXRα activation has been associated with increased liver fat deposition and the development of hepatic steatosis [17]. However, it has also been demonstrated to possess anti-inflammatory properties [18]. Furthermore, the reduction of LXRα produced a depletion of hepatic cellular lipid content in a fatty liver mouse model [19]. In contrast, other studies found that LXR activation improved liver injury, as evidenced by reductions in alanine aminotransferase (ALT), aminotransferase (AST), and tumor necrosis factor (TNF)α levels [20].

The aim of this study was to identify biomarkers in peripheral blood (a tissue easily obtained with minimally invasive techniques), which will enable us to gain a deeper understanding of how metabolic changes following RYGB may influence the relationship between leptin/LXRα pathways and different fat and fibrosis indexes. To this end, we analyze the mRNA expression of Ob-R, Sam68, LXRα, ABCA1, and ABCG1 in PBMCs from a group of non-obese subjects and patients with MO before and 6 months after RYGB.

## 2. Results

### 2.1. Anthropometric Characteristics and Biochemical Study

Table 1 summarizes the anthropometric and biochemical variables of non-obese subjects and patients with MO before and 6 months after RYGB. As expected, most of the anthropometric and biochemical parameters were altered in the obese group, which improved after RYGB, including fat (fatty liver index (FLI)) and fibrosis (non-alcoholic fatty liver disease fibrosis score (NAFLD FS)) indexes. Meanwhile, 0% of non-obese subjects had metabolic syndrome. In the MO group, 82% had metabolic syndrome before RYGB and 62.9% after RYGB.

### 2.2. LXRα, ABCA1, and ABCG1 Expression in PBMCs from Non-Obese Subjects and Patients with MO Pre- and Post-Surgery

**Figure 1:**
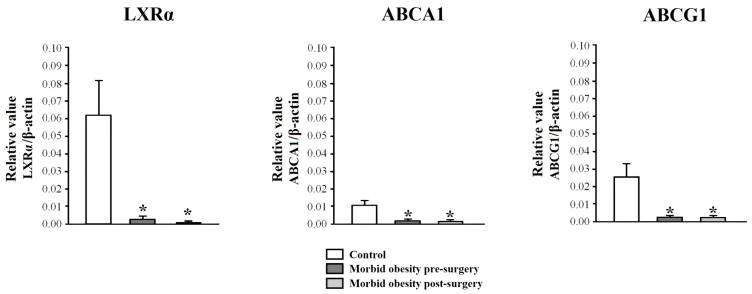
LXRα, ABCA1, and ABCG1 mRNA expression in PBMCs from non-obese subjects (control) and patients with morbid obesity pre- and post-surgery. Data are represented as mean ± standard error of the mean. * *p* < 0.001: significant differences with regard to control subjects.

Figure 1 shows the mRNA expression in PBMCs from non-obese subjects and patients with MO pre- and post-surgery. The mRNA expression of LXRα, ABCA1, and ABCG1 (*p* < 0.001, respectively) in PBMCs from patients with MO was statistically lower than in non-obese subjects. RYGB did not restore this gene expression. There were no significant differences in LXRα, ABCA1, and ABCG1 according to the presence or absence of metabolic syndrome.

### 2.3. Ob-Rb and Sam68 Expression in PBMC from Non-Obese Subjects and Patients with MO Pre- and Post-Surgery

**Figure 2:**
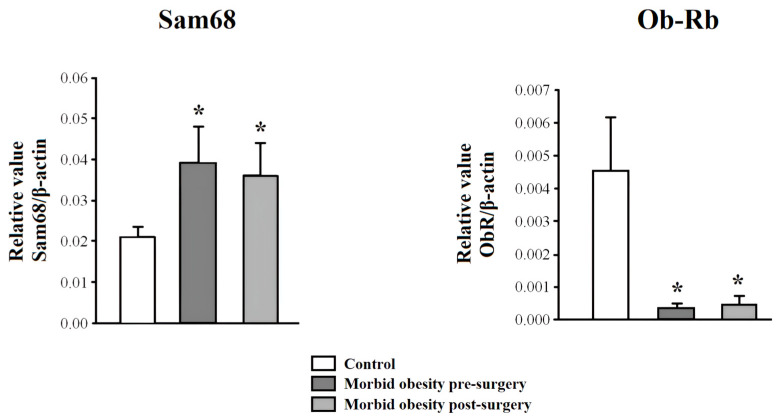
Ob-Rb and SAM68 mRNA expression in PBMCs from non-obese subjects (control) and patients with morbid obesity pre- and post-surgery. Data are represented as mean ± standard error of the mean. * *p* < 0.001: significant differences with regard to control subjects.

Figure 2 shows the mRNA expression in PBMCs from non-obese subjects and patients with MO pre- and post-surgery. Ob-Rb mRNA expression was significantly decreased (*p* < 0.001), and Sam68 expression was increased (*p* < 0.001) in PBMCs from MO patients compared to non-obese subjects. RYGB did not reverse this increased gene expression. There were no significant differences in Ob-Rb and Sam68 according to the presence or absence of metabolic syndrome.

### 2.4. Association between mRNA Expression and Anthropometric/Biochemical Variables in Patients with MO Pre- and Post-Surgery

**Figure 3:**
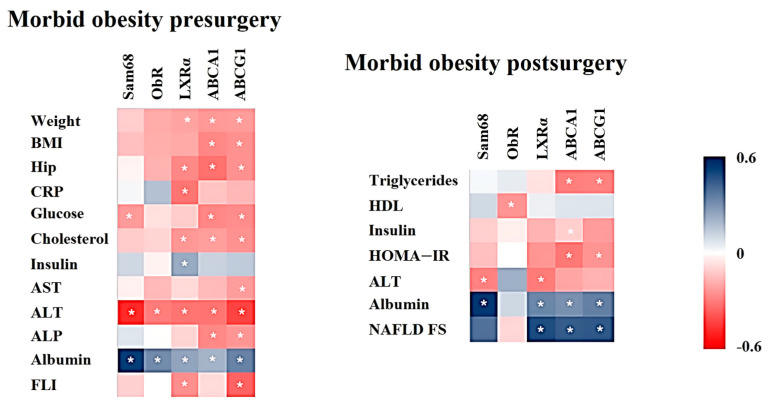
Heat maps represent those significant correlations between Ob-Rb, SAM68, LXRα, ABCA1, and ABCG1 mRNA expression in PBMCs from patients with morbid obesity pre- and post-surgery with anthropometric and biochemical variables. The Spearman correlation is displayed on a color scale from blue (positive correlation) to red (negative correlation). * Significant correlations (*p* < 0.05). BMI: body mass index; CRP: C-reactive protein; HDL: high-density lipoprotein; HOMA-IR: homeostasis model assessment of insulin resistance; AST: aspartate aminotransferase; ALT: alanine aminotransferase; ALP: alkaline phosphatase; FLI: fatty liver index; NAFLD FS: non-alcoholic fatty liver disease fibrosis score.

The mRNA expression in PBMCs from patients with MO before RYBGB was significantly correlated with several anthropometric (weight, BMI, and hip circumference) and biochemical variables (glucose, cholesterol, CRP, AST, ALT, alkaline phosphatase (ALP), albumin, insulin, and FLI) (Figure 3). Genes related to cholesterol transport, such as LXRα, ABCA1, and ABCG1, were those with a greater number of significant associations.

The mRNA expression in PBMCs from patients with MO six months after RYGB was significantly correlated with several biochemical variables, including triglycerides, HDL, ALT, albumin, NAFLD FS, insulin, and HOMA-IR (Figure 3). ABCA1 and ABCG1 were the genes with a greater number of significant associations.

## 3. Discussion

In the present work, we have extended the knowledge of metabolic changes on reverse cholesterol transport mechanisms and leptin response before and six months after RYGB as expected and as we have already shown in previous studies [21]. RYGB resulted in a reduction in peripheral inflammation (e.g., CRP levels) and T2DM. However, the recovery of lipid metabolism is slower and less understood. Although there was an improvement in HDL, the level was still lower than in the control subjects. Similar results have been previously described, although HDL levels were recovered after 12 months [22]. This improvement in HDL levels and its properties would have beneficial effects on obesity-associated atherogenic disease.

The mechanisms involved in the regulation of the atherogenic profile in patients with MO could include the ABCA1 and ABCG1 transporters. However, there are few studies on PBMCs. We found that the expression of ABCA1 and ABCG1 was reduced in patients with MO, as shown in another study performed in visceral adipose tissue [23]. This reduction could play an important role in the downregulation of HDL levels in MO. These two proteins are involved in the generation and formation of HDL [24,25]. Although previous studies have described that RYGB induces an improvement in the atherogenic lipid profile by a shift toward a more cardioprotective HDL and an increase in plasma efflux capacity via ABCG1 six months after surgery [26,27], our study did not show this increase. Our data at 6 months after RYGB suggest that, although there is a slight improvement, more time would be needed to normalize the expression levels of these genes in PBMCs and thus to normalize HDL levels and functionality.

In this study, we showed that LXRα expression was downregulated in PBMCs from patients with MO and negatively correlated with CRP and total cholesterol. In addition, LXRα, ABCA1, and ABCG1 expressions were negatively correlated with several obesity-related variables, such as weight, BMI, and hip circumference, reinforcing their association with obesity. Their reduced expression could be due to the increased serum LPS levels found in obesity [28], which repress the expression of LXR and, consequently, of ABCA1/ABCG1 [29]. Although this result may suggest that an upregulation of LXRα would be beneficial to increase the ABCA1 and ABCG1 expression, promote the reverse cholesterol transport (less total cholesterol), and reduce inflammation (less CRP), precautions must be taken. It is true that LXRα activation may suppress inflammation and improve atherosclerosis, but it can also promote the development of obesity and liver steatosis [30]

As expected, the high leptin levels found in patients with MO decreased to normal levels after RYGB. Although leptin can be secreted into the blood by a wide variety of tissues, in the case of morbid obesity, the most important tissues are the stomach and adipose tissue. The reduction in leptin levels after bariatric surgery is a result of the surgery itself due to the removal of part of the stomach and the large loss of adipose tissue. In previous studies, serum leptin levels were associated with an increased risk of sarcopenic obesity [31], suggesting that leptin may also play a role in sarcopenic obesity. However, it can deteriorate further after bariatric surgery [32]. The effects of leptin are mediated by its receptor, Ob-R, which was decreased in PBMCs from MO patients. Ob-Rb is critical because it is the only leptin receptor with a long cytoplasmic tail, allowing complete intracellular signaling. It plays a key role in mediating the effects of leptin on appetite control and energy balance via the JAK-STAT signaling pathway [33].

A previous study also showed that children with obesity had significantly lower Ob-R levels and higher leptin levels [34]. This decreased Ob-Rb expression could be due to the leptin excess and the low-grade inflammation present in obesity [35]. Regarding the effect of RYGB, although bariatric surgery has recently been described to enhance the leptin signaling pathway due to the decreased inflammatory state [36], our data did not show changes in mRNA expression. However, its level increased one year after laparoscopic sleeve gastrectomy, although it was still lower than that of the control group [37]. In addition, the weight loss one year after adjustable gastric banding increased soluble leptin receptor levels [38]. Perhaps more time would be needed to normalize its expression level. In the leptin pathway, we found an increase in the expression of Sam68. Sam68 influences alternative splicing of several genes critical to processes such as adipogenesis [39]. However, its obesity-related function in PBMCs has not yet been defined. In relation to bariatric surgery, the Sam68 gene still maintains high expression levels 6 months after RYGB. Further studies are needed to clarify the role of Sam68 in MO patients.

Another interesting point found in this study was the relationship between the expression of these genes and different liver variables. Although the gold standard for liver tests is liver biopsy, we did not have access to it for ethical reasons, nor to liver ultrasound or transient elastography. Therefore, we have used indirect markers, such as liver enzymes, FLI, and NAFLD FS, to study liver status. LXRα, ABCA1, and ABCG1 were negatively associated with ALT levels, which is related to lipid metabolism and obesity and reflects hepatocellular injury in patients with NAFLD [40,41]. Greater inhibition of LXRα, ABCA1, and ABCG1 would be associated with greater inflammation (CRP) and worse liver function (high ALT and AP levels and lower albumin concentration) [42]. Moreover, our data also showed that LXRα was associated with FLI, an indirect marker of liver steatosis, as suggested by a previous study [43]. The accumulation of fatty acids in the liver, as evidenced by elevated FLI levels, is a defining feature of MASLD [44]. This ectopic fat deposition is a consequence of the increased insulin resistance observed in obesity. In other organs, such as skeletal muscle, this fat deposition results in sarcopenia [45]. Furthermore, the severity of hepatic fibrosis and steatosis increases with the prevalence of sarcopenia [46]. Moreover, the relationship between sarcopenia and cholesterol metabolism is further supported by another previous study in which two proteins, cholesteryl ester transfer protein and apolipoprotein A2, were identified as potential biomarkers for sarcopenia, thereby improving the diagnostic accuracy of this condition [47]. On the other hand, the association found between Ob-Rb with ALT and albumin levels suggests that a decrease in leptin signaling may be associated with a hepatic impairment. In this regard, the development of NAFLD is associated with increased leptin levels and leptin resistance (low Ob-R signaling) [48]. In this regard, the up-regulation of leptin receptor may result in increased leptin sensitivity, which would be sufficient to treat NAFLD [49].

However, a limitation of the present study is that we performed an evaluation only 6 months after RYGB. At that time, the patient was still adapting to a new lifestyle and had not achieved total weight loss. In addition, it would have been better to conduct the study with liver samples. However, given the ethical impossibility of taking liver biopsies, we have carried out this study in PBMCs with the aim of finding biomarkers in peripheral blood (a tissue easily obtained with minimally invasive techniques). Other studies have also shown that changes in PBMC gene expression could be used as early biomarkers for the diagnosis of metabolic disorders, could have clinical significance, and could ultimately provide surrogate transcriptional markers of biological efficacy in relevant tissues [50,51].

In conclusion, this is the first time that it has been described that the expression of LXRα, ABCA1, ABCG1, and Ob-Rb in PBMCs was decreased in patients with MO before and after RYGB and was associated with several variables related to obesity, inflammation, and liver function. This decrease may play an important role in the downregulation of HDL levels and in the development of MASLD in MO. Our data at six months after RYGB suggest that more time would be needed to normalize the expression levels of genes related to reverse cholesterol transport or leptin responsiveness in PBMCs and thus to normalize HDL levels and liver function. Further research in this area is highly needed using transcriptomics and proteomics to perform a more complete analysis of the up- and down-regulation of specific obesity-related genes in these conditions, including in liver samples biopsied at the time of bariatric surgery.

## 4. Methods

### 4.1. Subjects

The study included 86 patients with MO before and six months after RYGB and 38 healthy, non-obese subjects (body mass index (BMI) ˂ 30 kg/m^2^). Patients were excluded if they had acute inflammatory disease, infectious disease, or because of patient decision. Non-obese subjects were similar in age to the group with MO, had a stable body weight for at least 3 months before the study, and without acute inflammatory disease, infectious disease, and lipid and carbohydrate metabolism disorders. The percentage of total weight loss (%TWL) was calculated as 100 × (baseline weight − postoperative weight)/baseline weight. The percentage of excess weight loss (%EWL) was calculated as 100 × [(baseline weight) − (postoperative weight)]/[(baseline weight) − (ideal weight)], where ideal weight is defined by the weight corresponding to a BMI of 25 kg/m^2^. The patients included in the study were subjected to the International Diabetes Federation (IDF) metabolic syndrome classification criteria to determine the percentage of patients who met the definition of presence or absence of metabolic syndrome [52]. The study was conducted in accordance with the Code of Ethics of the World Medical Association (Declaration of Helsinki). All participants gave their written informed consent, and the study was reviewed and approved by the Malaga Provincial Research Ethics Committee (Malaga, Spain).

### 4.2. Sample Collection

Peripheral blood samples were collected from patients with MO before and six months after RYGB and from non-obese subjects to isolate the cellular fraction and serum [53]. Serum was separated and immediately frozen at −80 °C. PBMCs were isolated from 10 mL of blood in a Ficoll-Paque density gradient (GE Healthcare, Buckinghamshire, UK) [53]. Samples were processed and frozen immediately upon receipt at the Regional University Hospital Biobank (Andalusian Public Health System Biobank, Spain).

### 4.3. Laboratory Measurements

Serum biochemical parameters were measured in duplicate using an Advia Chemistry XPT autoanalyzer (Siemens Healthcare Diagnostics, Malvern, PA, USA). The low-density lipoprotein (LDL) fraction was calculated according to the Friedewald equation. Serum insulin levels were measured by immunoassay using an ADVIA Centaur autoanalyzer (Siemens Healthcare Diagnostics, Malvern, PA, USA). The determination of leptin and high-sensitivity C-reactive protein (CRP) were performed by commercial enzyme-linked immunosorbent assay (Mediagnost GmbH, Reutlingen, Germany, and DRG Instruments GmbH, Marburg, Germany, respectively). HOMA-IR was calculated with the following equation: HOMA-IR = fasting insulin (µIU/mL) × fasting glucose (mmol/L)/22.5. The fatty liver index (FLI) was used for assessing hepatic steatosis [54]. To evaluate liver fibrosis, the NAFLD fibrosis score (NAFLD FS) was used [55].

### 4.4. Real-Time Quantitative PCR of mRNA Levels

Purification of RNA from PBMCs was performed by the QIAamp RNA Blood Mini Kit (QIAGEN Science, Hilden, Germany). Total RNA was reverse-transcribed into cDNA, and RT-PCR was carried out in an ABI Prism 7300 Sequence Detection System (Applied Biosystems, Foster City, CA, USA). PCR reactions were performed in triplicate with SYBR Green PCR Master Mix (Applied Biosystems, Foster City, CA, USA) using the following primers: LXRα: forward, 5’-AAGCCCTGCATGCCTACGT-3′, reverse, 5′-TGCAGACGCAGTGCAAACA-3′; ABCA1: forward, 5′-CCCTGTGGAATGTACCTATGTG-3′, reverse, 5′-GAGGTGTCCCAAAGATGCAA-3′; ABCG1: forward,5′-CAGTCGCTCCTTAGCACCA-3′, reverse, 5′-TCCATGCTCGGACTCTCTG-3′; Ob-Rb: forward, 5′-ATAGTTCAGTCACCAAGTGC-3′, reverse, 5′-GTCCTGGAGAACTCTGATGTCC-3′; Sam68: forward, 5′-TTTGTGGGGAAGATTCTTGG-3′, reverse 5′-GGGGGTCCAAAGACTTCAAT-3′ and β-actin: forward, 5’-CCAGCTCACCATGGATGATG-3’, reverse, 5’-ATGCCGGAGCCGTTGTC-3’. Relative levels of transcripts above were quantified by the comparative threshold cycle (Ct) method as described in the ABI Prism 7300 User Bulletin 2 and normalized to β-actin mRNA levels.

### 4.5. Statistical Analysis

Statistical analysis was performed using the SPSS 26.0 software package (SPSS Inc., Chicago, IL, USA). Normal distribution was analyzed using the Kolmogorov–Smirnov test. Most of the parameters analyzed did not have a normal distribution, and nonparametric analyses were used. Differences between two related variables were analyzed using the Wilcoxon test. The Spearman correlation coefficients were calculated to estimate the correlations between variables. Statistically significant differences were considered when *p* ≤ 0.05. Results are presented as mean ± standard deviation.

## Figures and Tables

**Table 1:** Anthropometric and biochemical variables of non-obese subjects and patients with morbid obesity (MO) before and six months after RYGB.

	Non-Obese Subjects	MO Pre-Surgery	MO Post-Surgery
Sex (Men/Women)	35 (12/23)	87 (22/65)	-
Age (years)	43.4 ± 13.9	42.5 ± 10.0	-
%EWL	-	-	36 ± 31.5
%TWL	-	-	17.3 ± 14.6
Weight (kg)	70.3 ± 13.0	135.3 ± 25.4 ^3^	96.5 ± 18.4 ^3,b^
BMI (kg/m^2^)	26.1 ± 3.9	50.4 ± 7.6 ^3^	36.1 ± 6.6 ^3,b^
Waist (cm)	92.5 ± 9.2	138.3 ± 16.2 ^3^	110.6 ± 13.1 ^3,b^
Hip (cm)	99.7 ± 8.0	147.5 ± 18.5 ^3^	121.6 ± 16.9 ^3,b^
Glucose (mg/dL)	85.7 ± 8.8	108.8 ± 49.6 ^3^	81.1 ± 13.5 ^b^
Cholesterol (mg/dL)	204.9 ± 30.6	197.3 ± 35.7	183.6 ± 33.5 ^2,b^
Triglycerides (mg/dL)	109.2 ± 61.3	149.7 ± 80.2 ^2^	109.9 ± 47.4 ^b^
HDL (mg/dL)	54.8 ± 12.1	47.2 ± 12.7 ^2^	47.0 ± 8.9 ^2,a^
LDL (mg/dL)	128.2 ± 27.0	120.0 ± 31.1	114.2 ± 30.3 ^1,b^
Insulin (µU/mL)	10.1 ± 4.5	22.0 ± 13.8 ^3^	8.8 ± 4.1 ^b^
HOMA-IR	2.17 ± 1.05	5.95 ± 4.5 ^3^	1.81 ± 0.88 ^b^
CRP (mg/dL)	3.3 ± 3.3	11.4 ± 7.0 ^3^	4.6 ± 4.1 ^b^
Leptin (ng/mL)	17.5 ± 12.7	71.1 ± 38.4 ^3^	19.28 ± 13.9 ^b^
Adiponectin (µg/mL)	10.7 ± 6.8	6.5 ± 3.3 ^2^	10.5 ± 5.5 ^c^
AST (IU/L)	22.0 ± 13.5	27.6 ± 18.6	20.9 ± 8.8 ^c^
ALT (IU/L)	25.5 ± 17.0	44.3 ± 33.1 ^3^	27.5 ± 14.5 ^c^
GGT (IU/L)	31.8 ± 32.2	38.4 ± 31.4	24.6 ± 26.3 ^2,c^
ALP (IU/L)	64.4 ± 30.1	79.5 ± 24.1 ^1^	75.1 ± 39.5 ^1^
Albumin (g/dL)	4.7 ± 0.3	4.0 ± 0.4 ^3^	4.0 ± 0.3 ^3,c^
FLI	53.5 ± 32.9	98.6 ± 2.9 ^3^	72.0 ± 25.7 ^2^
NAFLD FS	−1.17 ± 1.47	−0.69 ± 1.48	−1.53 ± 1.31 ^c^

Results are expressed as mean ± SD. Significant differences with respect to healthy control group: ^1^ *p* < 0.05, ^2^ *p* < 0.01, ^3^ *p* < 0.001. Significant differences in patients with MO between before and six months after RYGB: ^a^ *p* < 0.05, ^b^ *p* < 0.01, ^c^ *p* < 0.001. BMI: body mass index; HDL: high-density lipoprotein; LDL: low-density lipoprotein; HOMA-IR: homeostasis model assessment of insulin resistance; AST: aspartate aminotransferase; ALT: alanine aminotransferase; GGT: gamma-glutamyltransferase; ALP: alkaline phosphatase; FLI: fatty liver index; NAFLD FS: non-alcoholic fatty liver disease fibrosis score.

## Data Availability

The datasets presented in this article are not readily available because they are part of an ongoing study. Requests to access the datasets should be directed to the corresponding author, [E.G.-F.], upon reasonable request.
